# Smoking in patients with schizophrenia : “No smoking without fire”

**DOI:** 10.1192/j.eurpsy.2022.2031

**Published:** 2022-09-01

**Authors:** B. Abassi, E. Khelifa, K. Nourchene, I. Bouguerra, L. Mnif

**Affiliations:** 1 Razi, Skolly, Manouba, Tunisia; 2 Razi Hospital, F Adult Psychiatry Department, Manouba, Tunisia; 3 Razi hospital, Skolly, Tunis, Tunisia; 4 Errazi hospital-Mannouba , F, Mannouba, Tunisia

**Keywords:** schizophrénia, self-medication hypothesis, cigarettes, smoking

## Abstract

**Introduction:**

More than half patients with schizophrenia are smokers. Heavy smoking has been correlated to more severe positive symptoms, a higher number of hospitalizations and a less efficiency of antipsychotics. Unfortunately, abstinence is difficult to achieve in these patients, therefore it is importance of understanding the link between smoking and psychosis.

**Objectives:**

Analyzing the complex relationship between schizophrenia and nicotine’s effects on the human brain.

**Methods:**

The study was a review of literature over the past 10 years based on the pubmed database.

**Results:**

Smoking might be a precipitating factor in the development of schizophrenia since it preceded the onset of this illness for several years.Shared genetic background was also emphasized establishing a complex biological link between nicotine and schizophrenia.

In another approach, the “self-medication hypothesis” has been proposed suggesting a beneficial effect of nicotine on both cognitive impairment and negative symptoms in schizophrenia, related to the regulation of the dopamine and nicotinic receptor systems. But this conclusion is controversial since other studies concluded to a more neurocognitive impairment in smokers compared to controlled population.

**Conclusions:**

Smoking in schizophrenia is a complex “phenomenon” that remains, so far, misunderstood. Greater differences might exist between heavy and light smokers making it more difficult to point out the exact effect of nicotine on the brain. Smoking cessation therapies taking into account the specificity of patients with schizophrenia should be more developed.

**Disclosure:**

No significant relationships.

